# Traumatic aortic arch false aneurysm after blunt chest trauma in a motocross rider

**DOI:** 10.1186/1749-8090-3-23

**Published:** 2008-05-01

**Authors:** Federico Bizzarri, Consalvo Mattia, Massimo Ricci, Ilaria Chirichilli, Chiara Santo, David Rose, Luigi Muzzi, Giuseppe Pugliese, Giacomo Frati, Patrizio Sartini, Riccardo Ferrari, Carlo Della Rocca, Andrea Laghi

**Affiliations:** 1 Cardiac Surgery Unit, Department of Heart and Great Vessels "Attilio Reale", University of Rome "Sapienza", Polo Pontino, via F. Faggiana 34, 04100 Latina, Italy; 2 Department of Radiology, University of Rome "Sapienza", Polo Pontino, via F. Faggiana 34, 04100 Latina, Italy; 3 Department of Anatomy and Pathology, University of Rome "Sapienza", Polo Pontino, via F. Faggiana 34, 04100 Latina, Italy

## Abstract

This article details a case report of a traumatic aortic arch false aneurysm after blunt chest trauma. Thoracic aorta false aneurysms are a rare and life-threatening complication of aortic surgery, infection, genetic disorders and trauma.

## Background

Thoracic aorta false aneurysms are a rare and life-threatening complication of aortic surgery, infection, genetic disorders and trauma. After trauma approximately 2% to 5% of patients with aortic disruption develop a false aneurysm either after non operative treatment or lack of diagnosis [1]. Little is known about the natural history of this complication. However, a perfused false aneurysm may partially clot and organize with a fibrous wall potentially evolving into a saccular or fusiform aneurysm; late enlargement and even rupture may occur. Ninety percent of the false aneurysms involve the aortic isthmus; this probably reflects a sort of protection by the mediastinal periadventitial tissues at this level [2,3]. Patients developing chronic pseudoaneurysms show a low rate of associated injuries at the time of trauma [2,3]; in fact, 35% present no other injuries, and 50% only one.

## Case report

**Figure 1:**
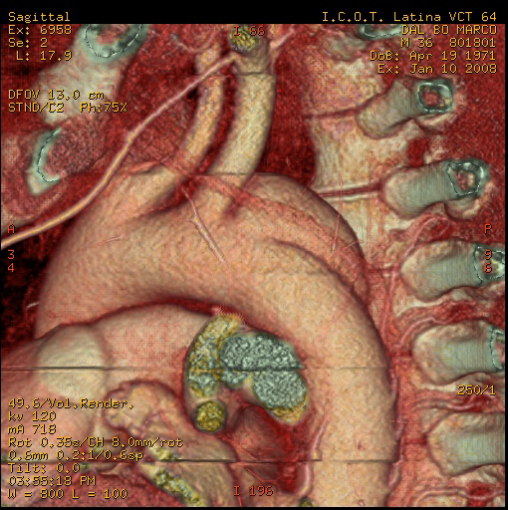
pseudoaneurysm and its relationship with surrounding structures.

**Figure 2:**
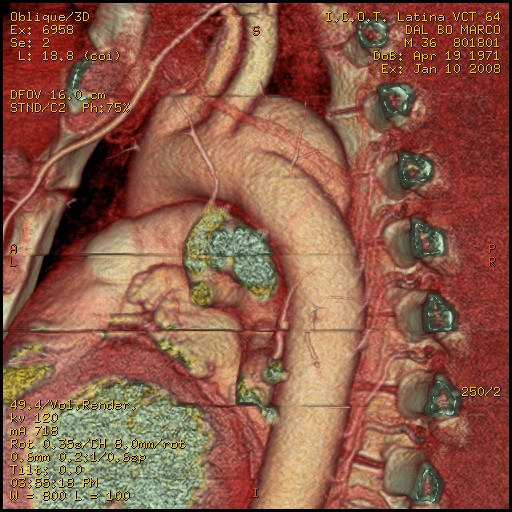
pseudoaneurysm and its relationship with vertebral spine.

**Figure 3:**
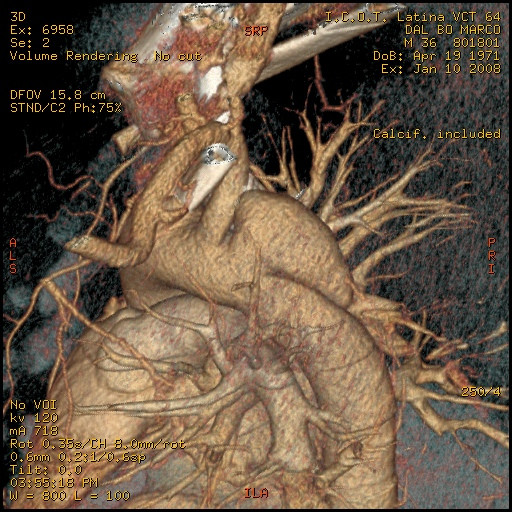
pseudoaneurysm and its relationship with subclavian artery.

A 33 year-old male motocross rider came to our attention complaining of back chest pain and cough. He referred a history of chest trauma 4 years ago during a motorbike race. The trauma resulted in an exstensive left shoulder and head injury associated to multiple rib fractures. He spent one month in hospital; he subsequently improved and was discharged in stable conditions. However, he continued to complain of a progressively increasing chest pain. At chest x-ray a left upper mediastinal mass was detected. A 64 multislice CT scan showed the presence of an aortic aneurysm (4 cm × 4.5 cm) arising from the descending thoracic aorta (Fig 1, 2, 3); the neck was located immediately after the origin of the left subclavian artery. on the convex aspect of the vessel. CT also showed the presence of a bovine configuration of the aorta. The diagnosis was "post-traumatic false aneurysm" involving the distal arch, as in most of the cases. The patient underwent endograft placement and fully recovered.
